# Supplementary material for: Cervical epithelial damage promotes Ureaplasma parvum ascending infection, intrauterine inflammation and preterm birth induction in mice
Source: Nat Commun. 2020 Jan 10;11:199. doi: 10.1038/s41467-019-14089-y (PMC6954262; doi:10.1038/s41467-019-14089-y)
Supplement: Supplementary file 1 — Supplementary Information [file 41467_2019_14089_MOESM1_ESM.pdf]

SUPPLEMENTARY INFORMATION FOR:

Cervical epithelial damage promotes *Ureaplasma parvum* ascending infection, intrauterine inflammation and preterm birth induction in mice.

Pavlidis *et al.*

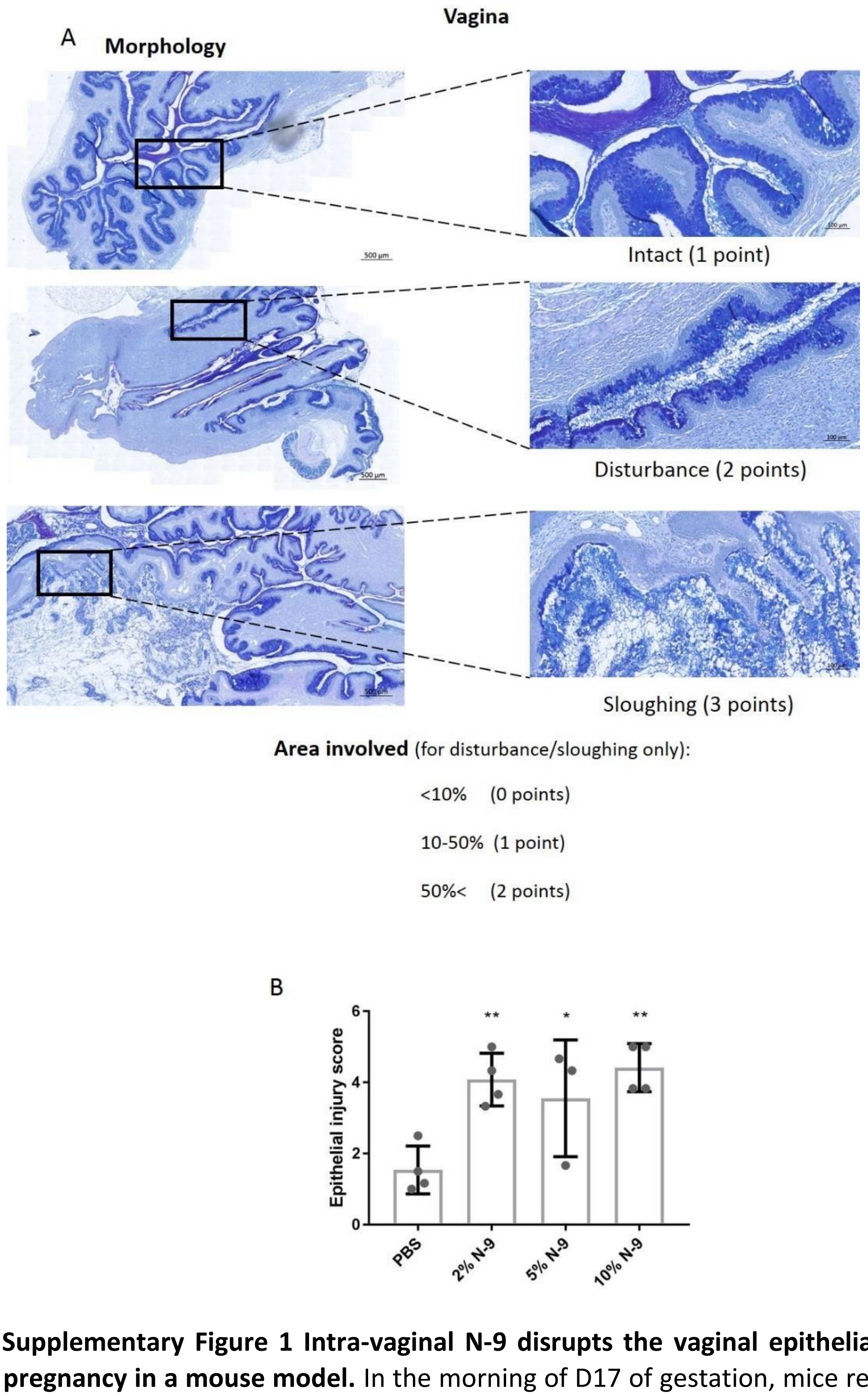

Supplementary Figure 1 Intra-vaginal N-9 disrupts the vaginal epithelial morphology during pregnancy in a mouse model. In the morning of D17 of gestation, mice received either N-9 (2% n=4, 5% n=3 or 10% n=4 in PBS) or PBS control (n=4) via intravaginal inoculation. 8 h later mice were sacrificed for tissue collections. Vaginal tissue sections were stained with AB/PAS and a morphological damage scoring system was used to assess epithelial damage (A). N-9 significantly damages the morphology of the vaginal epithelium during pregnancy (B). Error bars indicate *SD*. Statistical significance was assessed using 1-way ANOVA with Dunnett's multiple comparisons test against PBS group (**P<0.005 for 2% N-9 and 10% N-9 *vs.* PBS, *P<0.05 for 5% N-9 *vs.* PBS).

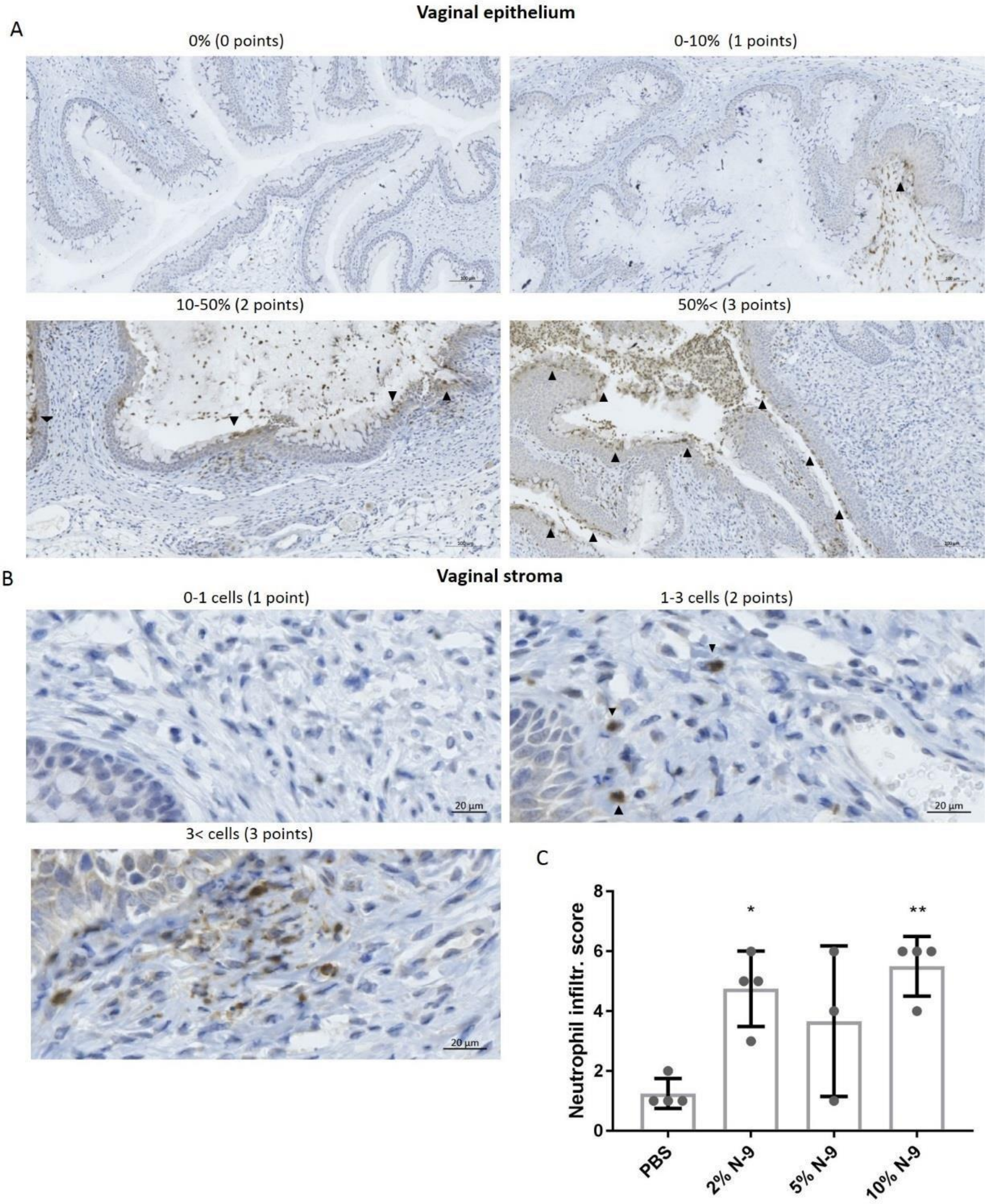

Supplementary Figure 2 Intra-vaginal N-9 results in polymorphonuclear neutrophils infiltrations in the vagina during pregnancy in a mouse model. In the morning of D17 of gestation, mice received either N-9 (2% n=4, 5% n=3 or 10% n=4 in PBS) or PBS control (n=4) via intravaginal inoculation. 8 h later mice were sacrificed for tissue collections. Anti-Ly6G immunohistochemistry on vaginal tissue sections was used to assess the presence of neutrophils. A neutrophil infiltration scoring system was used to quantify the presence of neutrophils in the vaginal epithelium (**A**) and stroma (**B**), the total score being the sum of the two. N-9 significantly increased the neutrophil infiltrations in the vagina (**C**). Error bars indicate *SD*. Statistical significance was assessed using 1-way ANOVA with Dunnett's multiple comparisons test against PBS group (**P<0.005 for 10% N-9 *vs.* PBS, *P<0.05 for 2% N-9 *vs.* PBS).

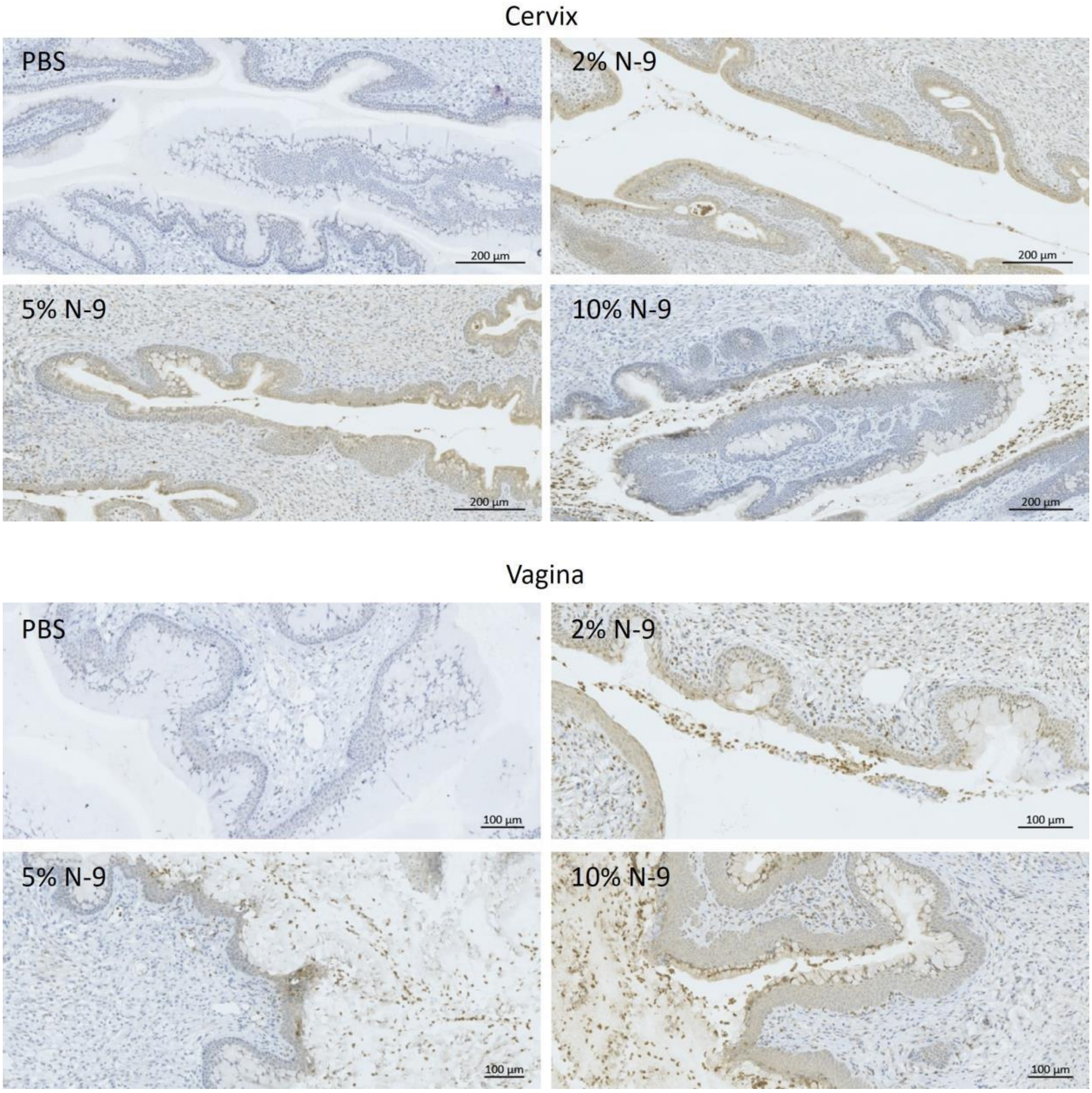

Supplementary Figure 3 Representative Ly6G IHC images from the different treatment groups. Showing neutrophil infiltration in different experimental groups.

Vagina

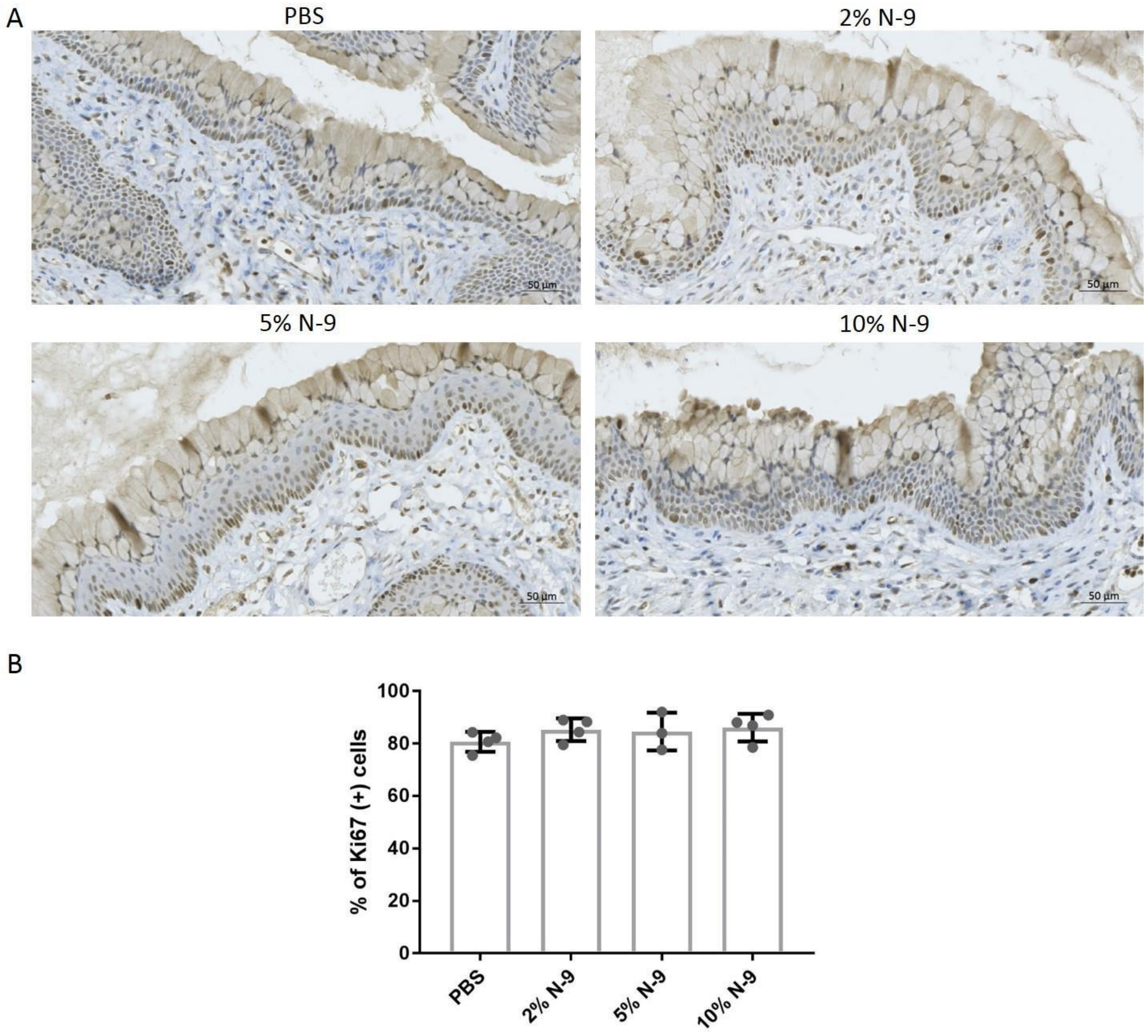

Supplementary Figure 4 Intra-vaginal N-9 has no effect on cell proliferation of the basal cells of the vaginal epithelium during pregnancy in a mouse model. In the morning of D17 of gestation, mice received either N-9 (2% n=4, 5% n=3 or 10% n=4 in PBS) or PBS control (n=4) via intravaginal inoculation. 8 h later mice were sacrificed for tissue collections. Anti-Ki67 immunohistochemistry on vaginal tissue sections was used to assess cellular proliferation at the basal layer of the vaginal epithelium. Representative images are shown (**A**). The percentage of Ki-67 positive cells was calculated across an area covering at least 1mm of the basal layer. N-9 has no effect on cellular proliferation at the vaginal basal layer (**B**). Error bars indicate *SD*. Statistical significance was assessed using 1-way ANOVA with Dunnett's multiple comparisons test against PBS group.

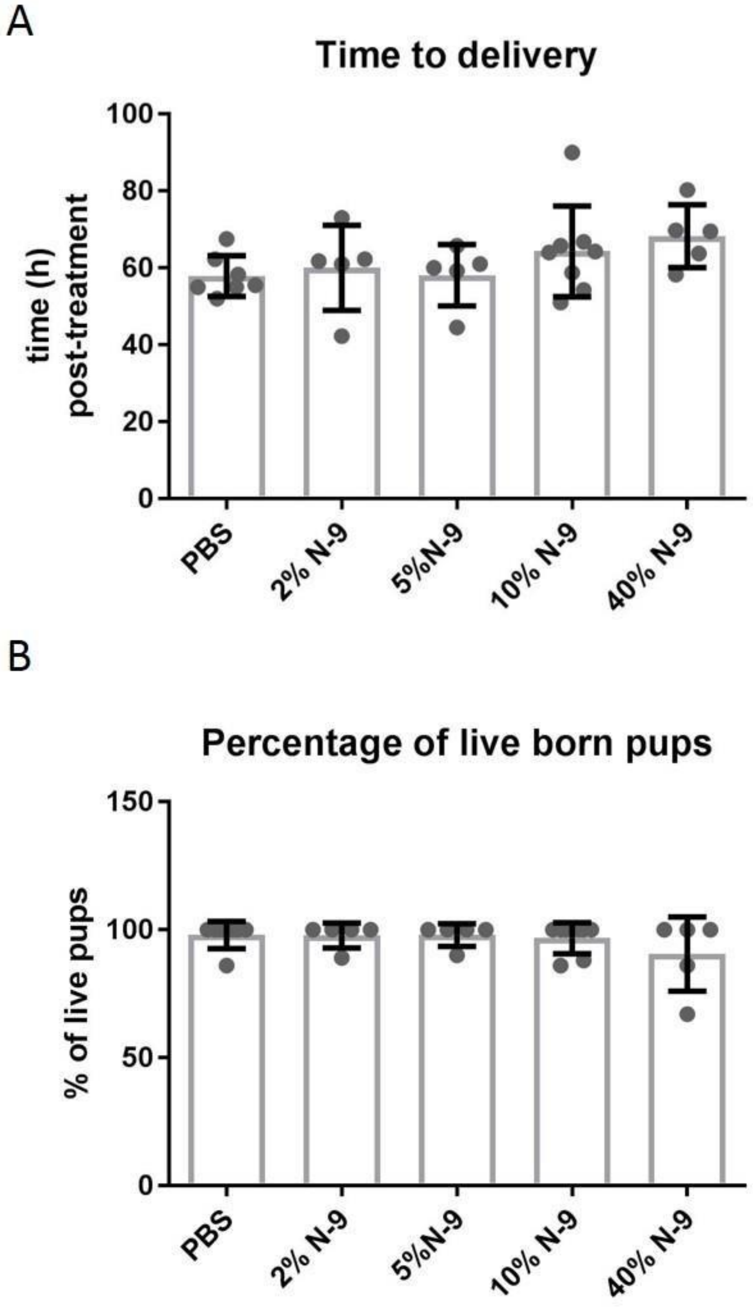

Supplementary Figure 5 Cervical damage caused by vaginal N-9 does not affect timing of delivery and pup survival in a mouse model of cervical damage during pregnancy. In the morning of D17 of gestation, mice received either N-9 (2% n=5, 5% n=5, 10% n=8 or 40% n=5 in PBS) or PBS control (n=7) via intravaginal inoculation. Time to delivery was calculated from the moment of intravaginal inoculation until the delivery of the first pup. N-9 had no effect on timing of delivery (1-way ANOVA with Dunnett's multiple comparisons test against 0% N9) (A). Percentage of live born pups for each mouse was calculated by dividing the number of live pups delivered by the total number of pups. N-9 had no effect on pup survival (1-way ANOVA with Dunnett's multiple comparisons test after arcsine transformation of proportions against 0% N-9) (B). Error bars represent *SD*.

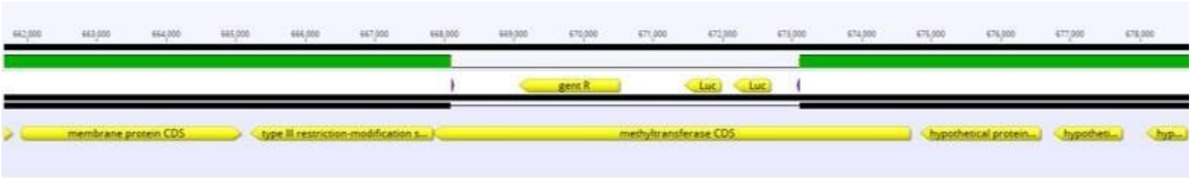

Supplementary Figure 6 Ureaplasma parvum mutagenesis. *Ureaplasma parvum* strain 137a1 (sub-strain of HPA5) was modified by site-directed mutagenesis following transformation with pMT85 as previously described (Aboklaish et al., 2014), with one modification: a codonoptimised sequence for Promega's Nanoluc® luciferase downstream from the *Ureaplasma tufA* promoter was synthesized by Genscript, and a tandem repeat was subcloned into the XbaI site in the pMT85 plasmid prior to delivery into the genome. Whole genome sequence assembly and analysis was performed by Geneious software (Version R10; Biomatters ltd. New Zealand) of the resultant modified strain subsequent to Illumina Nextera library preparation and processing by Illumina MiSeq sequencer. Geneious pairwise nucleotide alignment of genomes annotated relative to Ureaplasma parvum serovar 1 (accession number NZ_ABES00000000). Luciferase gene delivery into the genome was found to disrupt a non-essential methyltransferase gene (locus tag UPA1_G0351).

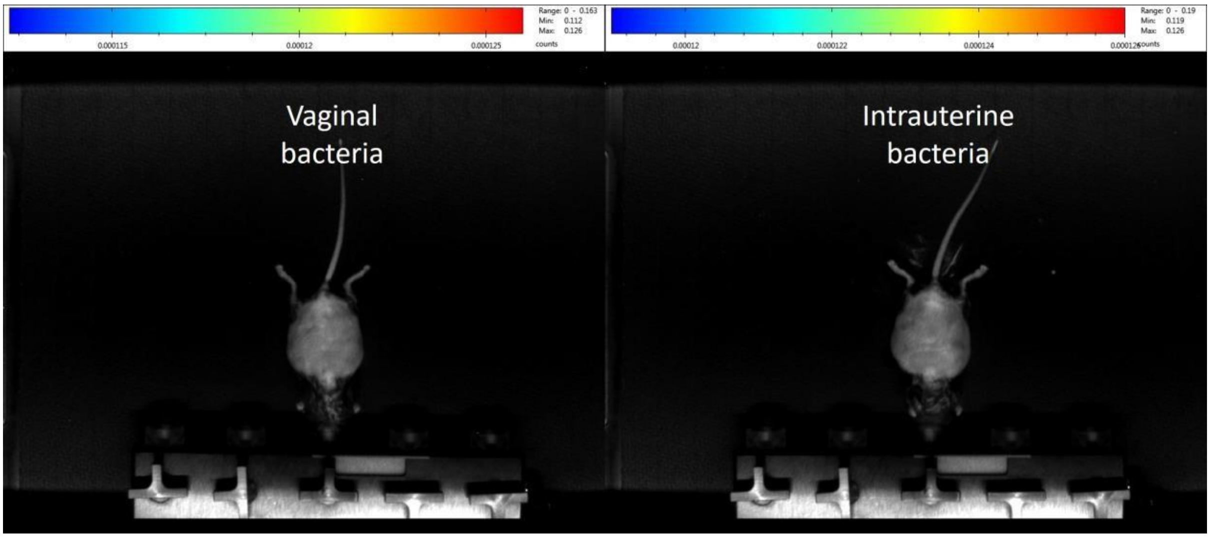

Supplementary Figure 7 Direct imaging of mice following intravaginal vs intrauterine administration of live UP+NanoLuc. Signal immediately following inoculation with luciferase-expressing *Ureaplasma parvum* from Supplementary Figure 8 (either vaginal – left or intrauterine by ultrasound-guided injection – right) was too weak to be detected by CCD camera.

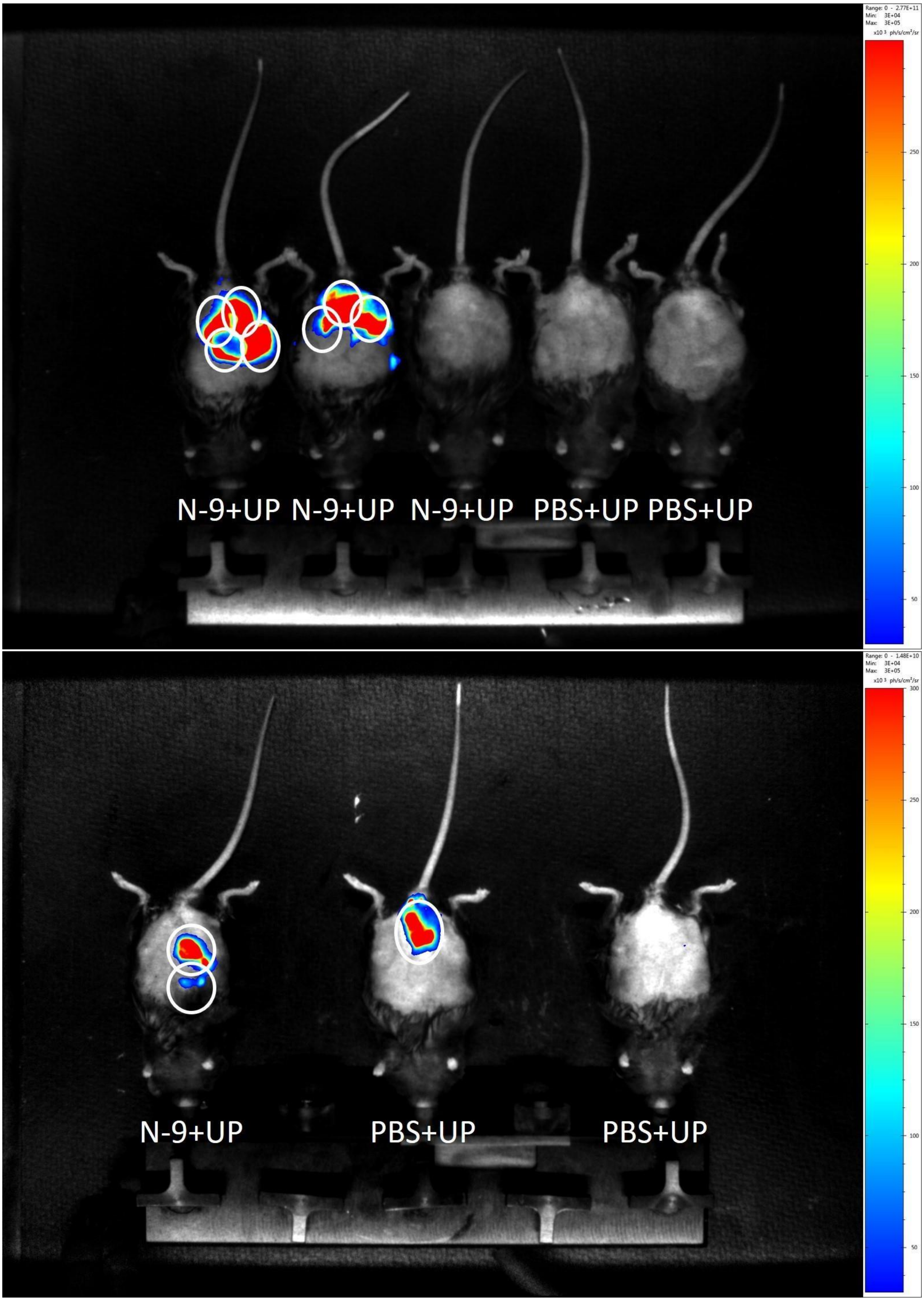
N-9+UP N-9+UP N-9+UP PBS+UP PBS+UP
N-9+UP
PBS+UP
PBS+UP

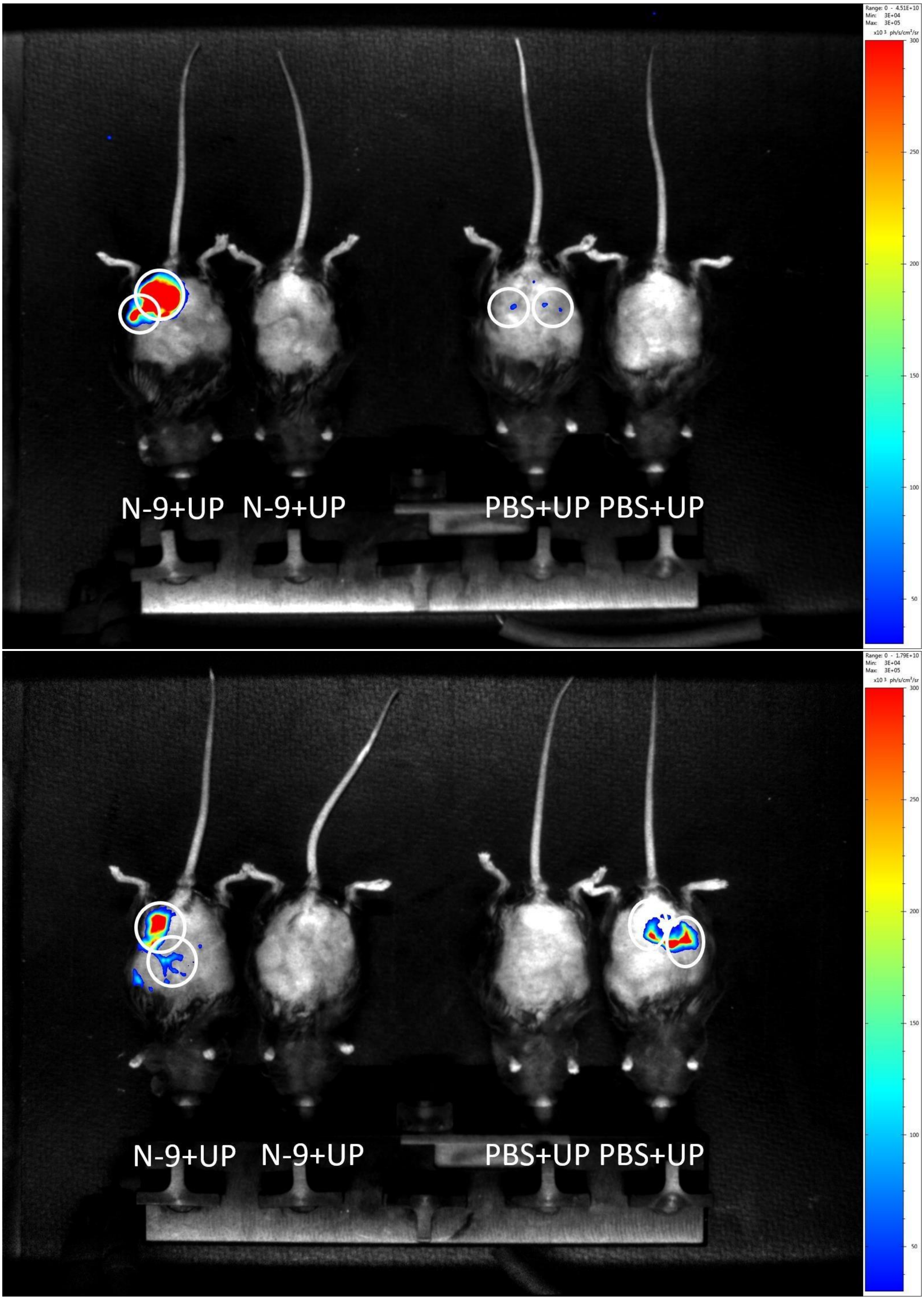

Supplementary Figure 8 Representative images depicting uterine bulges/gestating pups infected with UP following NanoLuc administration. Transabdominal imaging at 24 h postvaginal inoculation by luciferase-expressing *Ureaplasma parvum* was easily detected by CCD

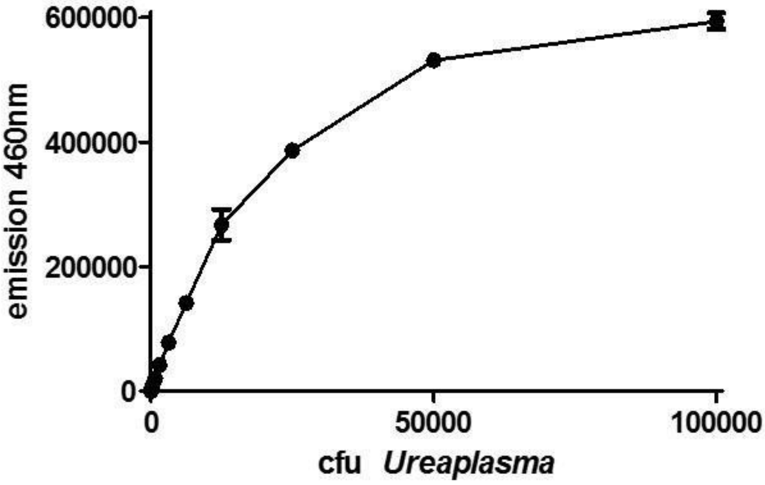

Supplementary Figure 9 *Ureaplasma parvum* bioluminescence. Bioluminescence by Luciferase-expressing strain HPA5 was determined by serial two-fold dilution in PBS of 100000 colony-forming units prior to addition of Promega Nano-Glo® live cell substrate (final volume of 100 µL) quantified by fluorimeter (BMG LUMIstar Galaxy) for a 30 second reading at emission 460 nm and reported in relative luminescence units. Readings were performed for triplicate dilutions with subtraction of PBS only controls. Luminescence is shown relative to amount of added bacteria, which was determined by titration in Ureaplasma selective media and enumeration on A7 (Biomerieux) agar plates after 48 hour incubation at 37°C. Curve analysis closely follows a non-linear one-site saturating binding kinetics, where 100 cfu HPA5luciferase was at the limit of detection from the top of an open 96-well plate. Ten-fold dilution of bacteria from 100,000 cfu reduced the signal by 80% and 100-fold dilution reduced the signal by 95%. Incubation of Ureaplasma with Promega Nano-Glo® Live Cell substrate did not reduce viability of the bacteria.

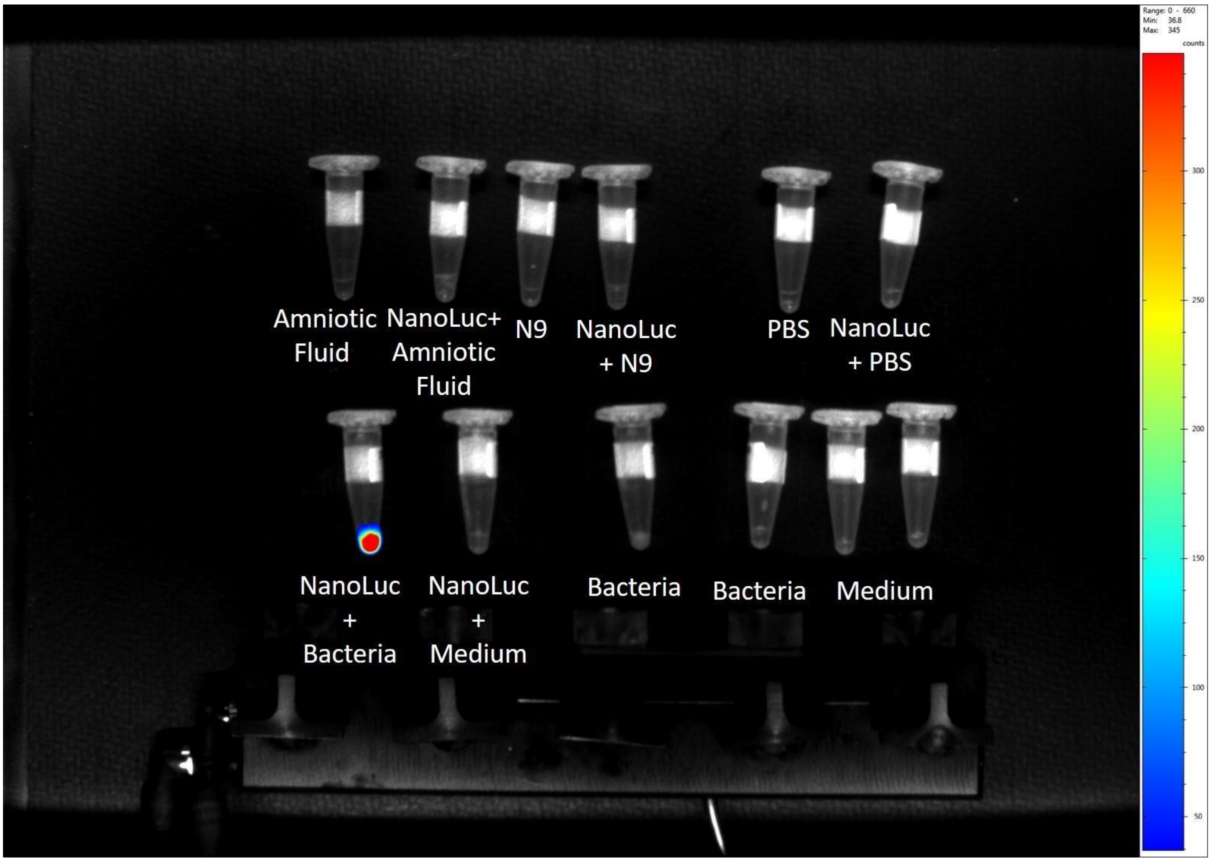

Supplementary Figure 10 Bioluminescent solutions and controls. White band on each tube represents the adhesive label – liquid volume in all tubes is that the bottom. NanoLuc labelling in this image represents addition of the NanoLuc substrate as all bacteria contained the NanoLuc gene.
